# Effects of Eicosapentaenoic Acid (EPA) and Docosahexaenoic Acid (DHA) on Fetal Pulmonary Circulation: An Experimental Study in Fetal Lambs

**DOI:** 10.3390/nu9070761

**Published:** 2017-07-16

**Authors:** Dyuti Sharma, Estelle Aubry, Thavarak Ouk, Ali Houeijeh, Véronique Houfflin-Debarge, Rémi Besson, Philippe Deruelle, Laurent Storme

**Affiliations:** 1 University Lille, CHU Lille, EA 4489, Perinatal Environment and Health, F-59000 Lille, France; dyuti.sharma@chru-lille.fr (D.S.); estelle.aubry@chru-lille.fr (E.A.); ali.houeijeh@chru-lille.fr (A.H.); veronique.debarge@chru-lille.fr (V.H.-D.); remi.besson@chru-lille.fr (R.B.); philippe.deruelle@chru-lille.fr (P.D.); 2 CHU Lille, Department of Paediatric Surgery, F-59000 Lille, France; 3 University Lille, Inserm, CHU Lille, U1171, Degenerative & Vascular Cognitive Disorders, F-59000 Lille, France; thavarak.ouk@univ-lille2.fr; 4 CHU Lille, Department of Neonatology, F-59000 Lille, France; 5 CHU Lille, Department of Obstetrics and Gynecology, F-59000 Lille, France

**Keywords:** polyunsaturated fatty acids, diet, persistent pulmonary hypertension of the newborn, prematurity, fetal pulmonary circulation

## Abstract

**Background:** Persistent pulmonary hypertension of the newborn (PPHN) causes significant morbidity and mortality in neonates. *n*-3 Poly-unsaturated fatty acids have vasodilatory properties in the perinatal lung. We studied the circulatory effects of eicosapentaenoic acid (EPA) and docosahexaenoic acid (DHA) in fetal sheep and in fetal pulmonary arterial rings. **Methods:** At 128 days of gestation, catheters were placed surgically in fetal systemic and pulmonary circulation, and a Doppler probe around the left pulmonary artery (LPA). Pulmonary arterial pressure and LPA flow were measured while infusing EPA or DHA for 120 min to the fetus, to compute pulmonary vascular resistance (PVR). The dose effects of EPA or DHA were studied in vascular rings pre-constricted with serotonin. Rings treated with EPA were separated into three groups: E+ (intact endothelium), E− (endothelium stripped) and LNA E+ (pretreatment of E+ rings with l-nitro-arginine). **Results:** EPA, but not DHA, induced a significant and prolonged 25% drop in PVR (*n* = 8, *p* < 0.001). Incubation of vascular rings with EPA (100 µM) caused a maximum relaxation of 60% in the E+ (*n* = 6), whereas vessel tone did not change in the E− (*n* = 6, *p* < 0.001). The vascular effects of EPA were significantly decreased in LNA E+ (*n* = 6). Incubation with DHA resulted in only a mild relaxation at the highest concentration of DHA (300 µM) compared to E+. **Conclusions:** EPA induces a sustained pulmonary vasodilatation in fetal lambs. This effect is endothelium- and dose-dependent and involves nitric oxide (NO) production. We speculate that EPA supplementation may improve pulmonary circulation in clinical conditions with PPHN.

## 1. Introduction

Many perinatal conditions such as premature rupture of the membrane, sepsis, meconium aspiration syndrome and pulmonary hypoplasia can promote cardiovascular maladaptation to extrauterine life in the newborn, called persistent pulmonary hypertension of the newborn (PPHN). This syndrome results from sustained elevation of pulmonary vascular resistance (PVR), causing severe hypoxemia. Chronic lung disease is associated with prolonged pulmonary arterial hypertension and may complicate the course of PPHN. These respiratory disorders occur in almost half of all infants weighing less than 1000 g at birth [1]. Currently, PPHN requires intensive treatments such as inhaled nitric oxide (NO) and inotropic agents, or invasive care such as extracorporeal membrane oxygenation (ECMO). Additional research is required that aims at decreasing occurrences of PPHN and its related lung morbidities.

Growing evidence indicates that the fetal nutritional environment plays a critical role in the maladaptation of the lung at birth. Differential vascular effects can be expected according to lipid intake. Many experimental, clinical and epidemiological studies have clearly demonstrated the beneficial role of dietary fish oils on cardiorespiratory function in adults. They reduce the occurrence of cardiovascular disease (atherosclerosis, myocardial infarction, hypertension) and improve survival following acute respiratory distress syndrome in adults [2,3]. Dietary fish oil enriched in *n*-3 PUFAs decreases lung vascular remodeling in rats with chronic hypoxia [4]. We have shown that, compared to soybean oil, fish oil has pulmonary vasodilatory properties in an experimental fetal sheep model [5]. *n*-3 PUFAs are found in fish oil, not in soybean oil. The aim of our study was to identify the active agent among the *n*-3 PUFAs responsible for these circulatory effects in the experimental fetal sheep model in vivo, and to test this agent in vitro on fetal pulmonary vascular rings to clarify its mechanism of action. Fish oil contains various *n*-3 PUFAs, mainly eicosapentaenoic acid (EPA) and docosahexaenoic acid (DHA).

## 2. Materials and Methods

### 2.1. Animals

All animal procedures and protocols used in this study were approved by the French “Ministère de l’Agriculture, de la Pêche et de l’Alimentation” before the studies were conducted, and were carried out in the Department of Experimental Research of our Hospital centre (animal experimentation agreement *n*°59286). Pregnant ewes of the Colombia–Rambouillet breed were housed in individual pens starting a week before (at 120 days of gestational age; total gestation duration: 145 days) and throughout the procedure.

### 2.2. Surgical Procedures

Fetal sheep underwent *in utero* surgery at a gestational age of 126 to 128 days, after fasting for 24 h. General anaesthesia was induced with intravenous propofol (Diprivan^®^, 5 mL, 2%; Astra Zeneca, Caponago, Italy) and maintained with isoflurane gas (Aerrane^®^, 1 to 5%; Baxter Healthcare Ltd., Norfolk, UK). Analgesia was provided by an injection of 20 mg of nalbuphine (Nubain^®^, 20 mg, Serb Laboratory, Paris, France).

After a midline laparotomy and hysterotomy, local anaesthesia (subcutaneous injection of 5 mL of lidocaine hydrochloride (10 mg/mL)) and fetal analgesia (intramuscular injection of 10 mg of nalbuphine) were performed in order to position catheters (18 G, Vygon^®^, Ecouen, France) in the fetal aorta and in the fetal superior vena cava through the axillary vessels. After a left thoracotomy, catheters were placed in the main pulmonary artery and the left pulmonary artery (LPA) (18 G and 21 G respectively), and an ultrasonic flowmeter probe size 6S (Transonic Systems Inc., Ithaca, NY, USA) around the LPA. During the closure of hysterotomy, an amniotic infusion of 250 mL of isotonic saline serum containing 500 mg of amoxicillin + clavulanic acid (Augmentin Intravenous, 1 g/200 mg, GlaxoSmithKline, Barnard Castle, UK) was carried out through a 16 G catheter left in intra-amniotic cavity to obtain a reference pressure. Postoperative analgesia of ewes was provided by an intravenous injection of 20 mg of nalbuphine, repeated 6 h after the first injection, and then on a daily basis until the third day after surgery. Similarly, antibiotic prophylaxis was administered by daily intra-amniotic injections of 500 mg of amoxicillin + clavulanic acid, and fetal analgesia provided by an intravenous injection of 10 mg of nalbuphine until the third day after surgery.

Catheters were maintained by daily injections of 2 mL of heparinized (10 IU/mL) normal saline, and their positions checked at autopsy. In vivo studies were performed after a recovery time of 48 h to obtain hemodynamic stability.

For in vitro experiments, laparotomy and hysterotomy sites were reopened at terms of 140 and 142 days, under general anaesthesia (with the same protocol as for surgery in vivo). The two fetal lungs, including the hila and main pulmonary artery, were removed by transverse thoracotomy using the technique as previously described [6], after local anaesthesia (5 mL of 1% lidocaine along the path of the incision) and fetal analgesia (intramuscular injection of 20 mg of nalbuphine). At the end of sampling, the lambs were euthanized using an intravenous injection of T61 S (Tanax^®^, Intervet, Beaucouzé, France) at a dose of 0.3 mL/kg.

### 2.3. Arterial Rings Preparation

The lungs collected were immediately immersed in Krebs solution (composition in mmol/L: NaCl 118, KCl 4.6, NaHCO_3_ 27.2, MgSO_4_ 1.2, KH_2_PO_4_ 1.2, CaCl_2_ 1.75, glucose 11.1, pH 7.35–7.45), maintained at 4 °C and oxygenated. Pulmonary arteries of the fourth generation from the main pulmonary artery were removed with caution to prepare 5 to 8 mm-long arterial rings (from one to six rings per fetus). Each ring was mounted by inserting a pair of calipers into the vascular lumen, and immersed in one of the four organ chambers (Radnoti^®^ Glass, Monrovia, CA, USA) containing 40 mL of Krebs solution maintained at 37 °C and oxygenated with a mixture 95% O_2_/5% CO_2_. In some rings, the endothelium was mechanically removed by gently rubbing with one caliper [7] before mounting them (E−). The lower caliper was fixed, and the upper caliper was suspended by a twine from a force transducer (Radnoti^®^, Covina, CA, USA) coupled to a computer card, Flash-12 (Strawberry Tree Incorporated, Sunnyvale, CA, USA). Dedicated software (Workbench^®^ PC, Strawberry Tree Incorporated, Sunnyvale, CA, USA) measured the contractile force of ring by changing the voltage between calipers. The tension of the pulmonary rings was very gradually adjusted to 0.7 g (optimal resting tension).

### 2.4. Physiological Measurements

#### 2.4.1. 1/In Vivo Protocol

Aortic, main pulmonary artery and intra-amniotic catheters were connected to a pressure-measuring monitor (Merlin, Hewlett-Packard, Palo Alto, CA, USA). Aortic pressure (PAo) and pulmonary arterial pressure (PAP) were referenced to the intra-amniotic pressure. Heart rate (HR) was calculated using the phasic signal from the PAP.

The left pulmonary artery (LPA) flow rate was continuously measured by the flowmeter using the mean of phasic blood flow signal, with zero blood flow defined as the measured flow value immediately before the beginning of systole. Pulmonary vascular resistance (PVR) was calculated as the difference between PAP and left atrial pressure divided by LPA flow, where left atrial pressure has been previously determined to be equal to intra-amniotic pressure + 2 cmH_2_O [8].

Blood samples from the main pulmonary artery were used for blood gas analysis (i-STAT analyzer, Abbott Laboratories, Abbott Park, IL, USA) and oxygen saturation measurement (OSM 3 hemoximeter and ABL 520 Radiometer, Copenhagen, Denmark).

#### 2.4.2. 2/In Vitro Protocol

Arterials rings were maintained at their optimal resting tension for 1 h. The viability was tested by the addition of 3 M KCl to obtain a final concentration of 80 mM. After several washes, pre-contraction was achieved by serotonin (5-HT, 100 µM). The reactivity of arterial rings was tested by endothelium-dependent relaxation in response to acetylcholine (ACh, 10 µM) with and without L-nitro-arginine (LNA), a specific NO synthase inhibitor. At the end of the protocol and after several washes with Krebs solution, a single dose of sodium nitroprusside (SNP, 10 µM) was applied after pre-contraction with 5-HT (100 µM) to test endothelium-independent relaxation (mediated by smooth muscle cells).

### 2.5. Experimental Design

#### 2.5.1. In Vivo Protocol: Fetal Pulmonary Haemodynamic Response to EPA, DHA or Ethanol Infusion

Prior to in vivo experimentation, a stable baseline was obtained with saline infusion (12 mL/h) for 30 min through the LPA catheter. Then, pulmonary haemodynamic response was studied during the infusion of EPA (25 mg), of DHA (50 mg) or their solvent (ethanol, 0.25 mL as a control) in saline solution into the LPA (12 mL/h) for 120 min, followed by saline infusion (12 mL/h) for one hour. After starting the infusion (T-20 min), EPA, DHA and ethanol had 20 min to reach the fetal circulation (T0) through the tubing. The dosage of EPA and DHA administered was chosen to match that in Omegaven^®^ (Fresenius Kabi, Sèvres, France) in the study of Houeijeh et al. [5]. Mean HR, mean PAP, mean PAo, mean intra-amniotic pressure and mean LPA blood flow were recorded at 10-min intervals. Blood gas and oxygen saturation measurements were performed at time 0 (start of EPA, DHA or ethanol infusion) and at 120 min (end of EPA, DHA or ethanol infusion).

#### 2.5.2. In Vitro Protocol: Vascular Response to EPA and to DHA in Fetal Pulmonary Artery Rings

This series of experiments was performed to determine the vascular response to EPA and to DHA in fetal pulmonary arterial rings pre-contracted with 5-HT (100 µM). Dose-response curves for relaxation were obtained by incrementally increasing the concentration of EPA or DHA (1–300 µM) once a plateau of contraction was reached using 5-HT.

Additional experiments were performed to explore the mechanism of EPA-induced pulmonary vasorelaxation. The vascular response to EPA was studied in fetal pulmonary arterial rings with (E+) or without endothelium (E−), pre-contracted with 5-HT (100 µM). Similar experiments were performed in fetal lamb pulmonary arterial rings with endothelium after incubation with L-nitro-arginine (100 µM) for 30 min (LNA E+). Dose-response curves for relaxation were obtained by incrementally increasing the concentration of EPA (1–300 µM) once a plateau of contraction was reached using 5-HT (100 µM). The responses of isolated artery rings to 10 µM ACh after pre-treatment with 100 µM LNA were assessed as controls.

### 2.6. Drugs Preparation

EPA (100 mg/mL) and DHA (200 mg/mL) were purchased from Cayman Chemical Laboratory (Ann Arbor, MI, USA). EPA (25 mg in 0.25 mL ethanol) and DHA (50 mg in 0.25 mL ethanol) were diluted in 23.75 mL of saline solution, and infused at a rate of 12 mL/h. In the control group, 0.25 mL ethanol (excipient of EPA or DHA) was diluted in 23.75 mL of saline, and infused at a rate of 12 mL/h.

All vasoactive drugs (ACh, 5-HT, SNP, KCl and LNA) were purchased from Sigma Chemical Co, Saint-Louis, MO, USA. ACh, 5-HT and SNP were made fresh daily as stock solutions of 0.1 M, and stored at 4 °C for the entire duration of the experiment. The LNA solution was also freshly prepared just before rings incubation. LNA (1 mg) was dissolved in a few drops of 1 M chlorhydric acid. Then, 40 mL of normal saline was added to obtain a 100 µM LNA solution. A few drops of 1 M sodium hydroxide was added to titrate the pH to 7.40.

### 2.7. Statistical Analysis

All statistical analyses were performed using StatView 4.5 software for PC (Abacus Concepts, Berkeley, CA, USA). For each protocol (in vivo and in vitro), n indicates the number of fetuses studied (independent measures).

Results are expressed as means ± SEMs or as the percentage of maximum variation from baseline. Intergroup comparisons of the longitudinal changes were analyzed with an analysis of covariance (ANOVA) and with the Bonferroni–Dunn test, using the baseline period (time −40 min to time 0) as the covariate, and the treatment period until 40 min after the end of treatment (time 0 to time 160 min) as the repeated observations. Blood gas parameters were compared using the non-parametric Wilcoxon test. A *p* value < 0.05 was considered statistically significant.

For in vitro protocols, one to six rings from each fetus were studied: the data were averaged to obtain a single value per fetus. ANOVA for repeated measures was used to analyze in vitro data. The vasorelaxant effect in vitro at 100 µM *n*-PUFA was compared between EPA and DHA groups using Mann and Whitney test.

## 3. Results

### 3.1. In Vivo Protocol: Pulmonary Haemodynamic Response to EPA (n = 8), DHA (n = 7) or Ethanol (n = 6) in Fetal Lambs

EPA did not alter HR (173 ± 2, baseline mean (T-40 to T0) vs. 175 ± 2, infusion mean (T0 to T160) beats/min; *p* = 0.87), PAP (52 ± 0.2, baseline mean, (T-40 to T0) vs. 52 ± 0.5, infusion mean (T0 to T160) mmHg; *p* = 0.55) or PAo (53 ± 0.4, baseline mean (T-40 to T0) vs. 51 ± 0.7, infusion mean (T0 to T160) mmHg; *p* = 0.41) over time. A significant increase in LPA blood flow was noted during EPA infusion, from 83 ± 0.32, baseline mean (T-40 to T0) to 100 ± 2 infusion mean (T0 to T160) mL/min (*p* < 0.001). The elevation of LPA flow was associated with a significant reduction in PVR from 0.52 ± 0.01 (baseline mean, (T-40 to T0) to 0.42 ± 0.01, infusion mean (T0 to T160) mmHg·mL^−1^ min (*p* < 0.001).

During the infusion of DHA, no statistical difference was observed in HR (164 ± 3, baseline mean (T-40 to T0) vs. 160 ± 3, infusion mean (T0 to T160) beats/min; *p* = 0.89), PAP (53 ± 0.5, baseline mean (T-40 to T0) vs. 54 ± 0.4, infusion mean (T0 to T160) mmHg; *p* = 0.54) or PAo (52 ± 0.5 vs. 52 ± 0.5 mmHg; *p* = 0.72). Mean LPA blood flow was not significantly different (84 ± 3 mL/min at baseline (T-40 to T0) and 89 ± 3, represented mean during DHA infusion (T0 to T160) mL/min (*p* = 0.157)). Similarly, changes in PVR were not significant (0.46 ± 0.03, baseline mean (T-40 to T0) mmHg·mL^−1^ min vs. 0.50 ± 0.03, infusion mean (T0 to T160) mmHg mL^−1^ min, *p* = 0.35).

**Figure 1:**
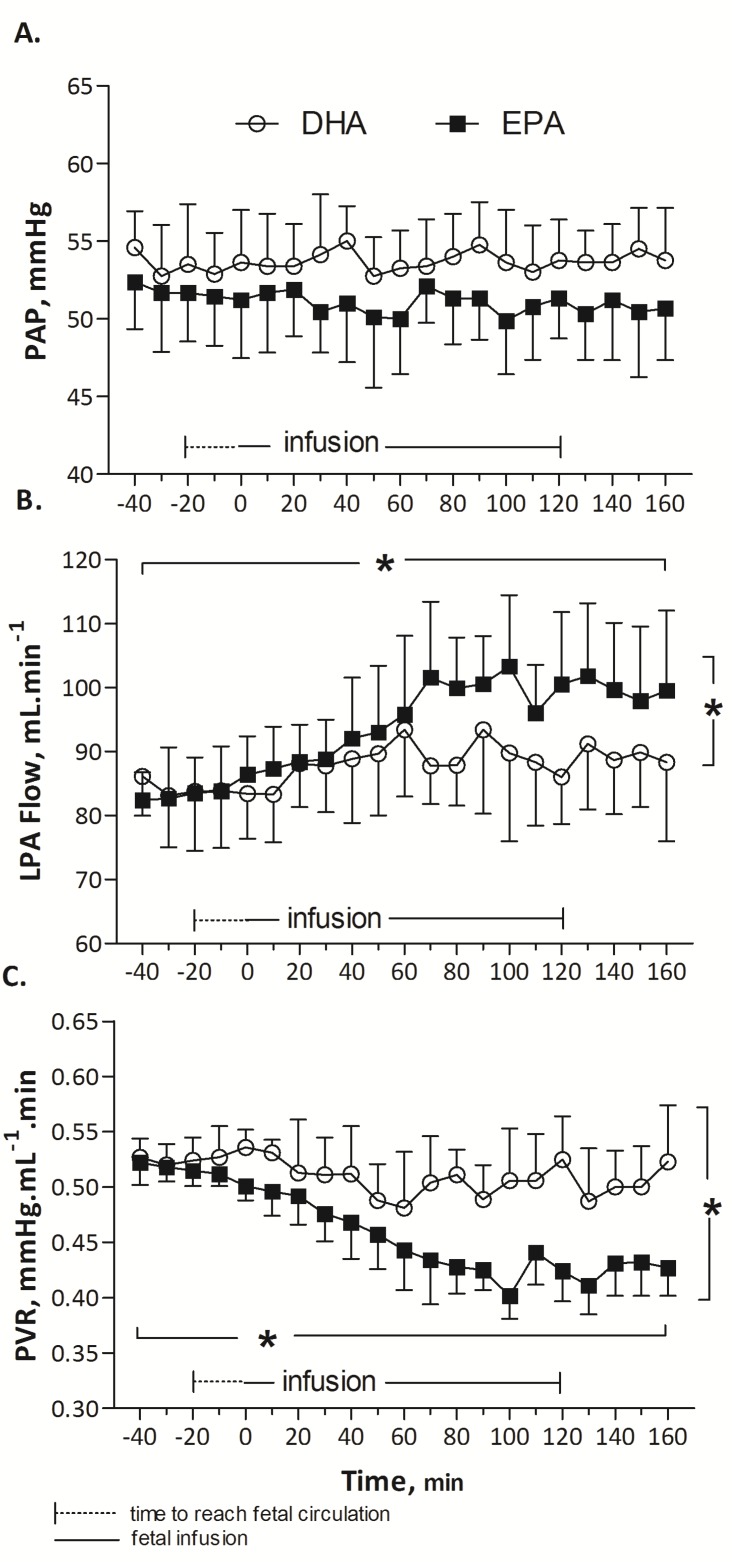
Evolution of pulmonary artery pressure, PAP (**A**), left pulmonary artery blood flow, LPA flow (**B**) and pulmonary vascular resistance, PVR (**C**) in response to infusion of eicosapentaenoic acid (EPA) compared to infusion of docosahexaenoic acid (DHA) (in vivo experiment). EPA or DHA were infused from T-20 to T120. LPA flow was greater (**B**) and PVR lower (**C**) during EPA than during DHA infusion. Values are means ± SEMs, *n* = 8 (EPA) or 7 (DHA). * *p* < 0.001 comparing EPA vs. DHA groups (Two-way ANOVA) and comparing baseline vs. EPA infusion (one-way ANOVA).

**Figure 2:**
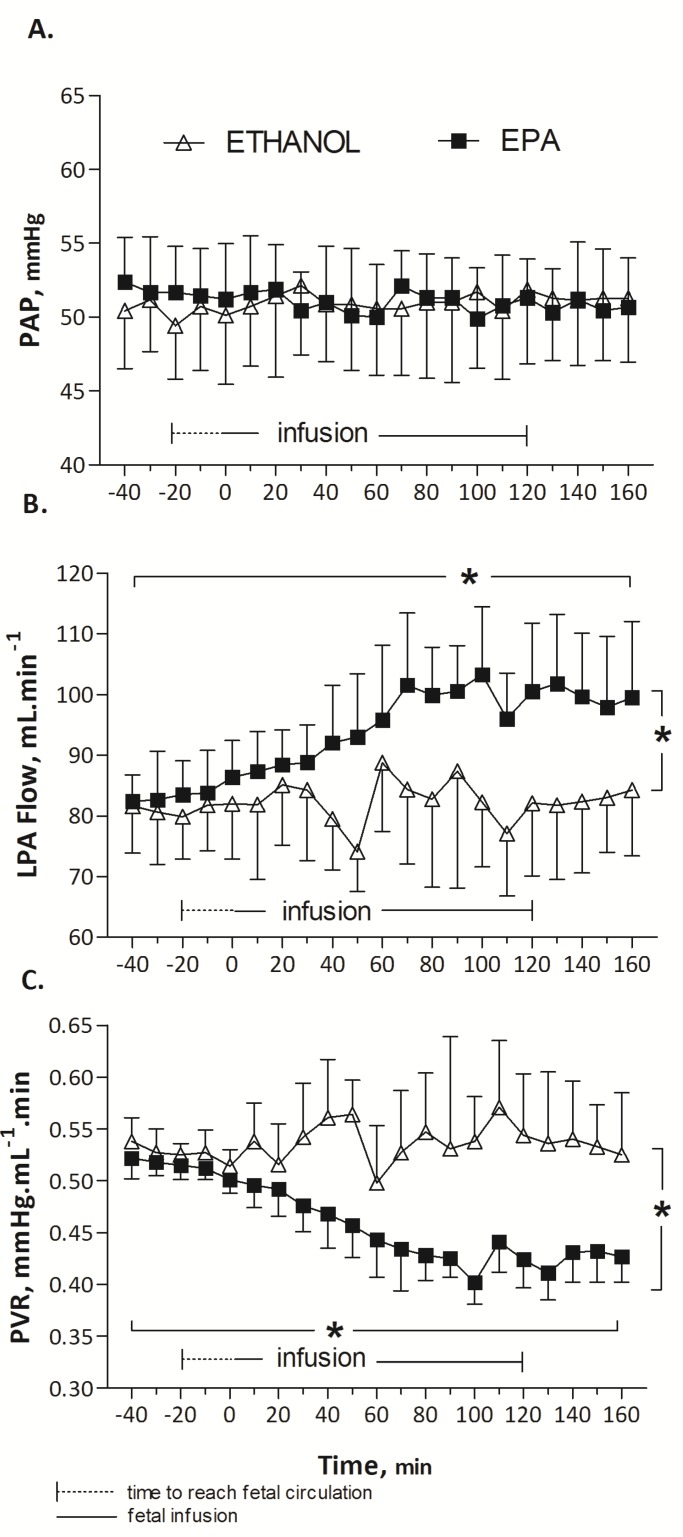
Evolution of pulmonary artery pressure, PAP (**A**), left pulmonary artery blood flow, LPA flow (**B**) and pulmonary vascular resistance, PVR (**C**) in response to infusion of EPA compared to infusion of ethanol (in vivo experiment). EPA or ethanol was infused from T-20 to T120. The LPA flow was greater (**B**) and PVR lower (**C**) during EPA than during ethanol infusion. Results are expressed as means ± SEMs, *n* = 8 (EPA) or 6 (control). * *p* < 0.001 comparing EPA vs. ethanol groups (Two-way ANOVA) and comparing baseline vs. EPA infusion (one-way ANOVA).

EPA induced a significant and prolonged 25% drop in pulmonary vascular resistance compared to DHA (*p* < 0.001, Figure 1) or ethanol (*p* < 0.001, Figure 2). Arterial blood gases, listed in Table 1, were not modified during EPA, DHA or control (ethanol) infusion (Wilcoxon test, *p* > 0.05). Infusion at a rate of 12 mL/h of 0.25 mL ethanol solution (control) did not significantly change HR (158 ± 0.6, baseline mean (T-40 to T0) vs. 159 ± 1.2 infusion mean (T0 to T160) beats/min, *p* = 0.75); PAo (49 ± 0.2, baseline mean (T-40 to T0) vs. 49 ± 0.1, infusion mean (T0 to T160) mmHg, *p* = 0.8), and pulmonary hemodynamic parameters: PAP (50 ± 0.3, baseline mean (T-40 to T0) vs. 51 ± 0.1, infusion mean (T0 to T160) mmHg, *p* = 0.18), LPA blood flow (81 ± 0.4, baseline mean (T-40 to T0) vs. 82 ± 0.8, infusion mean (T0 to T160) mL min^−1^, *p* = 0.12), and PVR (0.53 ± 0.003, baseline mean (T-40 to T0) vs. 0.54 ± 0.004, infusion mean (T0 to T160) mmHg mL^−1^ min, *p* = 0.30) (Figure 2).

### 3.2. In Vitro Protocol: Vascular Response to EPA and to DHA in Fetal Pulmonary Artery Rings

Ten lambs were sacrificed to carry out these in vitro protocols. Twenty-four rings were mounted. The E+ group (with endothelium) consisted of 12 rings and the E− group (stripped of endothelium) of six rings. Six rings with intact endothelium were incubated with LNA before pre-contraction with 5-HT, and represented the LNA E+ group.

All rings were contracted with 80 mM KCl to test vasoreactivity after mounting. The induction of relaxation with 10 µM ACh was higher in the E+ group compared to the E− group (respectively −20 ± 7% (E+ group, *n* = 6) vs. −5 ± 3% (E− group, *n* = 6), *p* < 0.05), confirming the presence or absence of endothelium. After pre-contraction with 100 µM 5HT, 10 µM ACh incubation caused a significant vasorelaxation by −45 ± 17%. The vasorelaxation induced by 10 µM ACh was significantly lower with 100 µM LNA than without LNA incubation (respectively +4 ± 11% (LNA+, *n* = 6) vs. −45 ± 17% (LNA-, *n* = 6), *p* < 0.001), indicating that ACh-induced vasorelaxation is mediated by NO production. Relaxation during incubation with 10 µM SNP was not significantly different between the E+ and E− groups (−50 ± 8%, E+ (*n* = 6) vs. −40 ± 5%, E− (*n* = 6), *p* = 0.20), indicating that the smooth muscle function was similar in the two groups at the end of the experiments.

**Figure 3:**
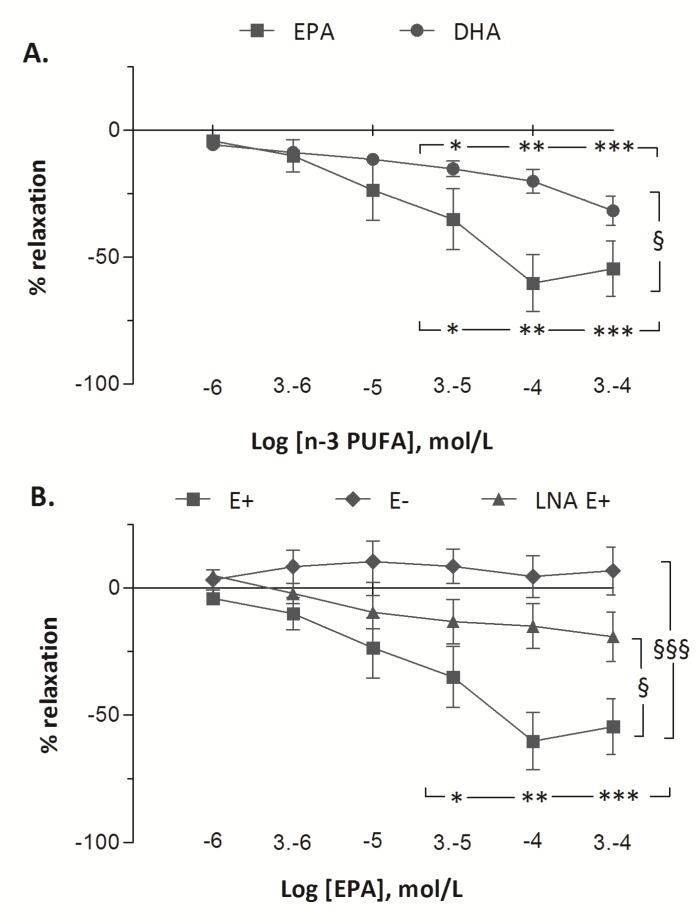
(**A**). Vascular response to incremental increase in EPA and DHA in fetal pulmonary artery rings with intact endothelium (E+ rings). After pre-contraction with 5-HT (100 µM), EPA causes a striking dose-response relaxation (−60% at 100 µM EPA), whereas a mild vasorelaxant response was observed from 30 µM (−15%) to 300 µM (−32%) of DHA. ^§^ p < 0.05 comparing EPA (*n* = 6) and DHA (*n* = 6) with Two-Way ANOVA and * *p* < 0.05, ** *p* < 0.01 and *** *p* < 0.001 compared to 1 µM EPA or DHA with One-Way ANOVA; (**B**). Vasoreactivity to EPA in pulmonary arterials rings with intact (E+, *n* = 6) or stripped endothelium (E−, *n* = 6), and after pre-incubation with L-nitro-arginine (LNA E+, *n* = 6) pre-contracted with 100 µM 5-HT. In the E− group, increasing concentrations of EPA did not change the rings tension. The vasorelaxant effect of EPA was lower in the LNA E+ group than in E+ group. Results are expressed as mean ± SEMs of % of variation of relaxation. ^§^ *p* < 0.05, and ^§§§^ *p* < 0.001 for E+ vs. E−, E+ vs. LNA E+ and * *p* < 0.05, ** *p* < 0.01 and *** *p* < 0.001 with one-way ANOVA for EPA.

The vascular responses to EPA and to DHA in fetal pulmonary artery rings are presented in Figure 3. Treatment with increasing doses of EPA and of DHA demonstrated vasorelaxant effects on fetal pulmonary arterial rings. Significant vasodilatation was observed from 30 µM of DHA to 300 µM (from −6 ± 2% at 1 µM to −15 ± 3% at 30 µM (*p* < 0.05) and to −20 ± 5% at 100 µM (*p* < 0.01) and to −32 ± 6 % at 300 µM (*p* < 0.001)). However, the vasorelaxant effect of EPA (from −4 ± 1% at 1 µM to −60 ± 11% at 100 µM, *p* < 0.001) was greater than DHA (*p* < 0.001). After pre-contraction with 5-HT (100 µM), incubation with increasing concentrations of EPA (1–300 µM) resulted in maximum relaxation in the E+ group (from −4 ± 1% at 1 µM to −60 ± 11% at 100 µM, *p* < 0.001). In the E− group, increasing concentrations of EPA did not change the tension of the rings (from +2 ± 1% to −1 ± 2%). The vasorelaxant effect of EPA was lower in LNA E+ group than in E+ group (maximum relaxation of 20% with LNA at 300 µM EPA) (*p* < 0.001). Although the vasorelaxant response to EPA was blunted when the vessel rings were pre-incubated with LNA, a significant dose-related vasorelaxant effect of EPA was still recorded (*p* < 0.05).

## 4. Discussion

In this study, we investigated the effects of *n*-3 PUFAs on the perinatal lung. We studied the effects of eicosapentaenoic acid (EPA) and docosahexaenoic acid (DHA) on basal pulmonary vascular tone in chronically monitored fetal lambs during the end of gestation. We found that the infusion of 25 mg of EPA induced a prolonged pulmonary vasodilatatory response, whereas the infusion of 50 mg of DHA did not change the pulmonary vascular tone. Increasing the dose of DHA in vivo should have raised the quantity of the solvent (ethanol) infused to fetal sheep. In ‘in vitro’ experiments, we observed a vasorelaxant effect in fetal lamb pulmonary arterial rings incubated with increasing doses of DHA. This effect was statistically significant from 30 µM to 300 µM of DHA. Using pulmonary arterial rings isolated from fetal lambs, we also showed that EPA induces a striking dose-related vasorelaxation. This vascular response to EPA is abolished by endothelial stripping and reduced by inhibition of endothelial NO synthase. Our study demonstrated that EPA and DHA have both vasodilatation properties, but EPA seemed to be more efficient than DHA to obtain vasodilatation on fetal pulmonary circulation.

Taken together, our data indicate that EPA causes a potent and sustained vasodilatation in the fetal pulmonary circulation, at least of half hour after the end of EPA infusion, through the activation of eNOS and enhanced production of NO.

Several studies have highlighted a protective cardiovascular effect of *n*-3 PUFAs, including EPA, in adults. A diet enriched in *n*-3 PUFAs reduces morbidity and mortality related to cardiovascular disease, both due to primary and secondary preventive effects [9,10]. Compared to diets containing *n*-6 PUFAs, *n*-3 PUFAs promotes endothelial function in healthy adults [11]. Other studies have investigated the effects of *n*-3 PUFAs on pulmonary circulation, especially in pathological conditions [12]. An experimental work in a rat model of chronic pulmonary hypertension has shown reduced mortality in hypoxic rats fed with fish oil (enriched in *n*-3 PUFAs) [4]. In an experimental rat model of pulmonary hypertension induced by monocrotaline injury, the inhibition of the degradation (by the soluble epoxide hydrolase) of *n*-3 PUFA-derived metabolites delays the vascular remodeling associated with pulmonary hypertension [13]. In the same way, the vasorelaxant effects of *n*-3 PUFA metabolites, including epoxy-eicosatrienoic acid, has been found in isolated human pulmonary arteries and bronchi [14,15]. The beneficial effects of *n*-3 PUFAs have also been described in adults with acute respiratory distress syndrome through a decrease in the production of pro-inflammatory mediators, and improvement of the endothelial function [16,17]. 

Our study provides new information on the effects of *n*-3 PUFAs on the perinatal lung. Eicosapentaenoic acid (EPA), but not docosahexaenoic acid (DHA), decreases pulmonary vascular resistance. This information indicates a potent vasodilatory effect of EPA within the lung, without adverse effect on the systemic haemodynamic, as aortic pressure and heart rate were not altered during EPA infusion. The vascular effect observed is also a direct effect of EPA, and not related to changes in oxygenation, as no significant changes in PaCO_2_ and the PaO_2_ are observed during EPA infusion. In addition, the absence of significant changes in blood gases and lactate indicate that placental perfusion and tissue oxygenation are not affected. Ethanol—in which EPA and DHA are dissolved—is not responsible for the vascular effects observed during the administration of EPA, as none of the hemodynamic parameters changed during ethanol infusion. In the present in vivo study, fetal lambs were infused at a rate of 3.6 mg kg^−1^ h^−1^ of EPA (i.e., 90 mg kg^−1^ day^−1^) and at a rate of 7.2 mg kg^−1^ h^−1^ of DHA (i.e., 170 mg kg^−1^ day^−1^). International guidelines for EPA and DHA requirements in the newborn infants range from 100 to 300 mg kg^−1^ day^−1^ containing around 1/3 EPA and 2/3 DHA [18]. The doses and concentrations of EPA and DHA used in our study are therefore clinically relevant.

Our in vitro experiments clearly highlighted a striking dose-related vasorelaxant effect of EPA in isolated fetal pulmonary vascular rings at concentrations ranging from 1 to 300 μM, i.e., from 0.3 µg/mL to 90 µg/mL. A range from 5 to 40 µg/mL (i.e., 100 µM) of plasma EPA and from 30 to 40 µg/mL (i.e., 125 µM) of plasma DHA concentrations have been previously measured in newborn infants receiving 3 g kg^−1^ day^−1^ parenteral fat emulsion enriched with 10% fish oil [19]. Another previous study indicates that dietary fish oil supplementation during pregnancy may result in a fivefold increase in EPA concentration and a twofold increase in DHA concentration in the plasma of the umbilical cord at birth, as well as in the plasma of newborn infants fed with breast milk whose mothers are supplemented with fish oil [20]. A recent review of literature studied the potential benefits of the supplementation of *n*-3 PUFA in childhood and adolescence to prevent cardiovascular disease and obesity [21]. In this study, the fetal (cord blood) and neonatal plasma concentrations of EPA (from 40 to 100 μM) are within the range of the maximal pulmonary vasorelaxant effects of EPA found in our in vitro study. At the opposite end of the range, only the highest concentration of DHA (300 μM) induced a slight pulmonary vessels’ relaxation. IV or dietary supplementation with fish oil results in an increase in plasma DHA concentrations from 75 to 125 μM [19,20]. In this range of DHA concentrations, significant vasorelaxant effects were observed in vitro, but were less potent than these obtained with EPA. Therefore, evidence exist that dietary supplementation with fish oil in pregnant or lactating women has the potential to elevate EPA and DHA concentrations in the fetus and the infant. However, it is likely that only EPA, but not DHA, may have the potential to alter the hemodynamics of the perinatal lung.

The mechanisms of action of *n*-3 PUFAs are complex and presently uncertain. In the current study, we addressed the mechanisms of action of EPA on isolated fetal pulmonary arteries. Endothelial damage abolishes the vasorelaxant effect of EPA, indicating that EPA-mediated relaxation is endothelium-dependent. Furthermore, the inhibition of eNOS by LNA significantly decreases the vasorelaxant effect of EPA. Previous studies [22,23] suggested the role of NO release in vascular response to EPA. In a study on bovine coronary arteries pre-contracted with PGF2α, EPA induced an endothelium-dependent vasorelaxant effect mediated by the calcium-independent activation and translocation of eNOS [22]. The relaxation induced by EPA on the pulmonary arteries of adult sheep in vitro has been associated with the release of NO by the endothelium [23]. However, our data in pulmonary vessels rings clearly highlights that a vasorelaxant response to EPA exists in spite of NO synthase inhibition, even though the response is reduced. Our previous study in fetal lambs is in accordance with this finding as a vasodilator effects of IV Omegaven^®^—a lipid emulsion enriched with polyunsaturated omega 3 fatty acids—was reported after NO synthase inhibition [5]. Taken together, these results indicate that the vascular effect of EPA in the perinatal lung is endothelium- and dose-dependent, and is mediated at least in part by endothelium NO synthase activation, but also by the release of other endothelium-dependent vasorelaxant mediators.

## 5. Conclusions

PPHN is characterized by the sustained elevation of pulmonary vascular resistance after birth. Medical management includes mechanical ventilation and inhaled NO. Our data provide evidence for potential beneficial effects of EPA on the pulmonary circulation. The amounts of EPA required for pharmacological effects are those provided by routine supplementation of fish oil-derived fat emulsion to parenteral nutrition. Parenteral nutrition supplemented with fish oil can be safely provided in neonatal care and improve outcomes in severe preterm neonates with respiratory distress syndrome [24]. We speculate that EPA supplementation may contribute to improved pulmonary circulation in PPHN.

Furthermore, several studies showed that the fetal nutritional environment plays a critical role in the maladaptation of the lung circulation at birth. For instance, it has been well established that maternal obesity and diabetes are associated with increased risks of PPHN [25]. In the same way, newborns with PPHN are deficient in the amino acid, L-arginine, which is required for NO synthesis [26]. Evidence exists that differential vascular effects can be expected according to lipid intake. Our study suggests that EPA may be beneficial in conditions associated with pulmonary hypertension through endothelial NO release. EPA can easily be transferred across the placenta toward the fetus after maternal intake of diet enriched in *n*-3 PUFAs [27], and also play a beneficial role in human milk composition, increasing alpha-linolenic acid [28]. We further speculate that dietary fish oils during the pregnancy may help to prevent maladaptation at birth in high risk conditions for PPHN.

## Figures and Tables

**Table 1:** Blood gases, hemoglobin saturation and plasma lactates concentration before and 120 min after starting infusion of eicosapentaenoic Acid (EPA, *n* = 8), docosahexaenoic Acid (DHA, *n* = 7) or control (ethanol, *n* = 6).

	EPA			DHA			Control (Ethanol)		
	Baseline	120 min after Starting EPA	*p*	Baseline	120 min after Starting DHA	*p*	Baseline	120 min after Starting Ethanol	*p*
**pH**	7.40 ± 0.04	7.38 ± 0.04	0.07	7.39 ± 0.07	7.38 ± 0.08	0.43	7.38 ± 0.05	7.36 ± 0.06	0.20
**pCO_2_, mmHg**	47 ± 7	48 ± 8	0.30	47 ± 5	46 ± 8	0.67	40 ± 6	40 ± 4	0.64
**pO_2_, mmHg**	18 ± 4	18 ± 4	0.53	18 ± 4	19 ± 5	0.06	15 ± 5	15 ± 4	1
**Lactates, mmol/L**	2 ± 1	2 ± 1	0.23	1 ± 1	2 ± 1	0.94	3 ± 5	4 ± 5	0.44
**HCO^3−^, mmol/L**	29 ± 4	28 ± 4	0.59	28 ± 3	27 ± 5	0.9	26 ± 7	23 ± 4	0.72
**HbO_2_, %**	50 ± 20	52 ± 18	0.55	52 ± 19	54 ± 21	0.25	60 ± 18	64 ± 25	0.38

Baseline values compared to values measured 120 min after starting EPA or DHA by Wilcoxon test. Values are means ± SEMs, significant at *p* < 0.05.

## Abbreviations

ACh	Acetylcholine
DA	ductus arteriosus
DHA	docosahexaenoic acid
ECMO	extracorporeal membrane oxygenation
eNOS	endothelial nitric oxide synthase
EPA	eicosapentaenoic acid
E+	rings with endothelium intact
E−	rings with endothelium stripped
5-HT	serotonin; HR, heart rate
LNA	l-nitro-arginine
LNA E+	rings pre-treated with LNA and with endothelium intact
LPA	left pulmonary artery
NO	nitric oxide
*n*-3 PUFAs	*n*-3 polyunsaturated fatty acids
PAo	aortic pressure
PAP	pulmonary arterial pressure
PPHN	persistent pulmonary hypertension of the newborn
PVR	pulmonary vascular resistance; SNP, sodium nitroprusside
